# Spatially resolved transcriptomics revealed local invasion-related genes in colorectal cancer

**DOI:** 10.3389/fonc.2023.1089090

**Published:** 2023-02-01

**Authors:** Hong-Tao Liu, Si-Yuan Chen, Ling-Long Peng, Li Zhong, Li Zhou, Si-Qi Liao, Zhi-Ji Chen, Qing-Liang Wang, Song He, Zhi-Hang Zhou

**Affiliations:** ^1^ Department of Gastroenterology, the Second Affiliated Hospital of Chongqing Medical University, Chongqing, China; ^2^ Centre for Lipid Research & Key Laboratory of Molecular Biology for Infectious Diseases (Ministry of Education), Institute for Viral Hepatitis, Department of Infectious Diseases, The Second Affiliated Hospital, Chongqing Medical University, Chongqing, China; ^3^ Department of Gastrointestinal Surgery, the Second Affiliated Hospital of Chongqing Medical University, Chongqing, China; ^4^ Department of Pathology, the Second Affiliated Hospital of Chongqing Medical University, Chongqing, China

**Keywords:** colorectal cancer, metastasis, prognosis, heterogeneity, spatial transcriptomics

## Abstract

**Objective:**

Local invasion is the first step of metastasis, the main cause of colorectal cancer (CRC)-related death. Recent studies have revealed extensive intertumoral and intratumoral heterogeneity. Here, we focused on revealing local invasion-related genes in CRC.

**Methods:**

We used spatial transcriptomic techniques to study the process of local invasion in four CRC tissues. First, we compared the pre-cancerous, cancer center, and invasive margin in one section (S115) and used pseudo-time analysis to reveal the differentiation trajectories from cancer center to invasive margin. Next, we performed immunohistochemical staining for RPL5, STC1, AKR1B1, CD47, and HLA-A on CRC samples. Moreover, we knocked down AKR1B1 in CRC cell lines and performed CCK-8, wound healing, and transwell assays to assess cell proliferation, migration, and invasion.

**Results:**

We demonstrated that 13 genes were overexpressed in invasive clusters, among which the expression of CSTB and TM4SF1 was correlated with poor PFS in CRC patients. The ribosome pathway was increased, while the antigen processing and presentation pathway was decreased along CRC progression. RPL5 was upregulated, while HLA-A was downregulated along cancer invasion in CRC samples. Pseudo-time analysis revealed that STC1, AKR1B1, SIRPA, C4orf3, EDNRA, CES1, PRRX1, EMP1, PPIB, PLTP, SULF2, and EGFL6 were unpregulated along the trajectories. Immunohistochemic3al staining showed the expression of STC1, AKR1B1, and CD47 was increased along cancer invasion in CRC samples. Knockdown of AKR1B1 inhibited CRC cells’ proliferation, migration, and invasion.

**Conclusions:**

We revealed the spatial heterogeneity within CRC tissues and uncovered some novel genes that were associated with CRC invasion.

## 1 Introduction

The reconstruction of the cellular architecture, functional orientation, and evolutionary trajectory of cancer have been achieved by multi-omic studies over the past decade. It is well accepted that most tumors are complicated ecosystems that emerge and evolve under selective pressure from the tumor microenvironment, which promotes the diversification of both malignant and nonmalignant components. All these processes resulted in intertumoral heterogeneity and intratumoral heterogeneity (ITH) that enables aggressive disease progression and resistance to treatment (1). This ITH, including spatial heterogeneity, is featured with intratumoral distinct clones as a consequence of evolutionary processes.

Recent single-cell omics studies, especially single-cell RNA sequencing (scRNA-seq) technology, have greatly advanced our understanding of the tumor heterogeneities (2), tumor-infiltrated immune cell subpopulations (3), and the features of tumor-associated stromal cells (4) at single cell level. Although these studies provided extensive novel insights into tumor heterogeneity, the scRNA-seq technology still has limitations, especially losing the spatial and morphologic information. The recently developed spatial transcriptomics (ST) technology could overcome the above limitations with high throughput (5). ST provides high-quality genome-wide transcriptome with intact two-dimensional positional information by positioning histological cryosections on arrayed reverse transcription primers with unique positional barcodes (6). It has been applied to analyze the spatial heterogeneity of human primary liver cancer (5, 7), melanoma (8), ovarian carcinoma (9), breast cancer (10), prostate cancer (11), and pancreatic ductal adenocarcinomas (12). However, more studies are needed to fully reveal the spatial architecture of tumor tissues.

Colorectal cancer (CRC) is the third most common cancer in men and the second most common cancer in women worldwide (13). It has been estimated that there will be about 1.9 million new cases and 1 million deaths in 2020 (14). The major contributor to CRC-related death is distant metastasis (15). Approximately 25% of patients present with metastatic foci at initial diagnosis, and almost half of CRC patients ultimately develop metastases (16). Tumor metastasis is a multi-step cascade. As the first step of metastasis, local invasion of CRC occurs from the epithelial layer to serosal layer (17). The depth of invasion is associated with advanced tumor stage and poor prognosis (18). Previous studies have demonstrated that the epithelial-to-mesenchymal transition (EMT) promotes cancer cell local invasion (19). However, the cancer progression is so complex that even the EMT process is dynamically changing. The spatial heterogeneity along the CRC invasion path is not fully known.

10× Genomics Visium spatial transcriptome sequencing can correlate the gene expression patterns in different regions and their spatial distribution through high-throughput *in situ* detection of frozen sections (20). Thus, we can directly observe differences in gene expression between different functional regions of the tissue. We herein applied this technology in four CRC tissues from 4 patients to reveal the ITH along the invasion direction. We uncovered some novel genes that were associated with CRC invasion and progression. The expression of STC1, AKR1B1, RPL5, and CD47 was increased, while HLA-A was downregulated along cancer invasion by immunohistochemical staining in clinical samples. We also revealed that knockdown of AKR1B1 could inhibit the proliferation, migration, and invasion of CRC cells. These results highlighted the intratumoral heterogeneity within CRC tissues.

## 2 Materials and methods

### 2.1 Sample collection and preparation

Fresh tissues were collected from the resected tissues of four patients with colorectal cancer, preferably 10 mm × 8 mm × 6 mm in size. Excess fluid, such as blood, was aspirated using dust-free paper (Kimtech, Cat#: 0131-10, Texas, USA) to ensure that as many tissues as possible were of no liquid residue. The prepared tissues were put into the mold (Leica, Cat#:14702218313, 6mm×8mm, Hesse, Germany) after marking the direction from epithelial layer to serosal layer using forceps. A little pre-cooled OCT (pre-cooled at four °C for ≥30 min; Sakura, Cat#: 4583, CA, USA) was then injected into the mold to thoroughly cover the tissue. Finally, the embedded tissue was transferred to a -80°C freezer for storage.

We collected resected tissues from 45 CRC patients in the Second Affiliated Hospital of Chongqing Medical University. Immunohistochemical staining of PRL5, HLA-A, STC1, AKR1B1, and CD47 expression was performed on tumor tissue sections. The study was approved by the ethical review board of the Second Affiliated Hospital of Chongqing Medical University (Chongqing China; Project identification code: 2019.133) and all patients signed informed written consent.

### 2.2 Tissue processing and Visium data generation

OTC-embedded tissues were sectioned and imaged with HE staining to optimize whether the section covers the target region. Tissue sections were placed on the slides containing RNA-binding capture probes. After fixation and permeabilization, the mRNA in the cells was released and bound to the corresponding capture probes. cDNA synthesis and sequencing library preparation were performed using the captured RNA as a template. The Next Generation Sequencing was performed on the prepared sequencing libraries. HE results were combined to determine the spatial location information.

### 2.3 Original data processing

Samples were initially processed using the Space Ranger software provided by 10× Genomics. Space Ranger uses an image processing algorithm to show the captured areas of tissue in the microarray and distinguish each Spot’s reads based on spatial barcode information. The total number of spots, the number of pair reads in each spot, the number of genes detected, and the number of UMIs were also counted using the genomic matching software STAT to assess the quality of the samples.

### 2.4 Dimensionality reduction and clustering

Principal Components Analysis (PCA) was used for dimensionality reduction, and t-SNE was used for clustering. Since the visualization result of t-SNE clustering showed a significant distinction between samples of spot groups in each sample slice, we switched to the Mutual Nearest neighbors (MNN) algorithm for batch effect removal.

### 2.5 Cell type identification

Using SPOTlight, a cell type identification software was explicitly developed for 10X Visium technology. A non-negative matrix decomposition (NMF) based deconvolution algorithm was used to infer the cell composition of each spot by combining single-cell transcriptome data (sc-RNAseq) and cell type marker gene information with spatial transcriptome data.

### 2.6 Identification of spatial characteristic genes

The absolute number of genes within a spot was obtained using measured transcript sequences combined with UMI and spot barcode. Counting the proportion of mitochondrial genes, the number of gene expressions, and the number of UMIs in a single spot shows the sample quality. We used sctransform in Seurat software to normalize the data and construct a negative binomial model of gene expression to detect high variance features. Using Seurat software, differential expression analysis was performed based on pre-labeled regions within the tissue, which can be determined by unsupervised clustering or prior knowledge and ultimately by the findmarker function.

### 2.7 Gene set variation analysis

GSVA is a non-parametric unsupervised analysis method. The gene set corresponding to each pathway was first calculated as a rank statistic similar to the K-S test. The expression matrix was converted into a pathway enrichment scoring (ES) matrix to get a GSVA enrichment score for each pathway corresponding to each cell. The pathways with significant differences were then obtained using limma package analysis to assess the degree of enrichment of different pathways among different groups.

### 2.8 Survival and gene expression analysis

Survival analysis was performed using the cSurvial website (https://tau.cmmt.ubc.ca/cSurvival/) (21). This website provides users with an integrated database including TCGA, and analysis tools for gene prognostic assessment. P-values <0.05 were considered statistically significant. After opening the website, we selected TCGA-COAD or TCGA-READ and entered the time of censored cases at 5 years in the top panel. In the middle panel, we selected the option of progress-free interval. In the bottom panel, we selected the option of ‘‘gene or location’’ and expression, and input the target gene to get the result.

Gene expression analysis was performed using the UALCAN website (http://ualcan.path.uab.edu/). This website is mainly based on the relevant cancer data in the TCGA database for analysis and provides users with tools for gene expression analysis. First, we selected TCGA analysis, entered the target gene and cancer type on the upper right panel, and started the analysis. Then select the option of gene expression and get the results based on the Individual cancer stages.

### 2.9 Cell trajectory and pseudo-time analysis

The pseudo-time analysis is also known as cell trajectory analysis. Using the Monocle software package, the dynamics of temporal development were simulated by first using the expression patterns of critical genes for machine learning. The genes with considerable intercellular variation in gene expression were selected. Their expression profiles were spatially downscale to construct a minimum spanning tree (MST), which was then used to find the differentiation trajectory of cells with similar transcriptional characteristics by the longest path.

### 2.10 Immunohistochemical staining

Sections were deparaffinized, hydrated, and incubated in 3% hydrogen peroxide for 10 min, then washed three times for 3 minutes each in PBS (pH 7). Put the sections into sodium citrate buffer (pH 6.0), heated them in the microwave twice for ten minutes, and set them aside to cool at room temperature. and washed three times in PBS (pH 7.4) for 3 minutes each. According to the size of the tissue, drop an appropriate amount of primary antibody, incubate at 4°C overnight, washed three times for 3 minutes each in PBS. Antibodies used include STC1 (1:50; Proteintech, China), RPL5(1:800; Proteintech, China), AKR1B1(1:50; BOSTER, China), HLA-A(1:100; BOSTER, China) and CD47(1:400; BOSTER, China). Add an appropriate amount of enhanced enzyme-labeled goat anti-mouse/rabbit IgG polymer dropwise, incubate at room temperature for 20 minutes, washed three times for 3 minutes in PBS each. Use freshly prepared DAB chromogenic solution for visualization, rinse with PBS, and then counterstained with hematoxylin staining solution. The IHC results were viewed by an independent pathologist and whole section scanning was done using Pannoramic Scan (3DHISTECH, Budapest, Hungary).

### 2.11 Cell culture

Human colorectal cancer cell lines (SW620, HCT116, LoVo, HT29, and SW480) were obtained from the American Type Culture Collection (Manassas, VA, USA). Cells were cultured in Dulbecco’s modified Eagle’s medium (DMEM; Gibco, Gaithersburg, MD, USA) added with 10% fetal bovine serum (Gibco, Rockville, MD, USA) and 1% penicillin-streptomycin (NCM Biotech, Suzhou, CHN). All cells were cultured in a humidified 5% CO2 environment.

### 2.12 Lentivirus infection

Lentiviruses carrying small hairpin RNA (shRNA) sequences of human AKR1B1 were purchased from Obio Company (Shanghai, China). For infection, SW620 cells and HCT116 cells were plated into 12-well plates and incubated for one day. Then these cells were infected with control, shAKR1B1-1, shAKR1B1-2, or shAKR1B1-3 lentivirus at a multiplicity of infection (MOI) of 3. Sequences for AKR1B1 shRNA and control were as follows: shRNA-1 (5’-CCAGGTGGAGATGATCTTAAA-3’); shRNA-2 (5’-GCCTGCAGTTAACCAGATTGA-3’); shRNA-3 (5’-TGCTGAGAACTTTAAGGTCTT-3’); control (5’-CCTAAGGTTAAGTCGCCCTCG-3’). After 18 hours of infection, the culture medium containing lentiviral was removed, and fresh culture medium containing 10% fetal bovine serum was added to the culture plate to continue the culture. The positive selection was performed by adding puromycin (2µg/ml; Solarbio, Beijing, China).

### 2.13 RNA extraction and real-time quantitative PCR

Total RNA was extracted from each group with TRIzol Reagent (Takara, Dalian, China). Complementary DNA (cDNA) was subsequently synthesized using PrimeScript RT Master Mix (Takara, Japan). The qPCR was performed using cDNA (50ng/reaction) by CFX96 real-time PCR system (Bio-Rad, Hercules, CA, USA) with SYBR^®^ Premix Ex TaqTM II kit (Takara, Japan). The primer sequences used are as follows: GAPDH: 5’- GCACCGTCAAGGCTGAGAAC -3’(forward), 5’- TGGTGAAGACGCCAGTGGA -3’ (reverse); AKR1B1: 5’- ACGCATTGCTGAGAACTTTAAG -3’(forward), 5’- TTCCTGTTGTAGCTGAGTAAGG -3’ (reverse).

### 2.14 Western blotting

Whole-cell protein was extracted using a protein lysis buffer(Beyotime, Shanghai, China).25μg samples were loaded in the well and were separated by 10% SDS-PAGE and transferred to polyvinylidene fluoride membranes (Millipore, Billerica, MA, USA). Then blocked with 5% nonfat milk powder in TBST (TBS with 0.1% Tween) for 2 hours at room temperature and incubated with the appropriate primary antibody at 4°C overnight. Antibodies used include AKR1B1(1:50; BOSTER, Wuhan, China) and β-tubulin(1:4000; Abmart, Shanghai, China). The next day these membranes were washed with TBST (TBS with 0.1% Tween) three times for 5 minutes each. Then membranes were incubated with HRP-coupled anti-rabbit secondary antibodies (1:1000; ZSGB-BIO, Beijing, China) and anti-mouse secondary antibodies (1:1000; ZSGB-BIO, Beijing, China) at room temperature for 2 hours. Next, membranes were washed with TBST (TBS with 0.1% Tween) three times for 5 minutes each. Finally use ECL reagents (Beyotime, Shanghai, China) and visualize by an enhanced chemiluminescence detection system (Thermo Scientific, Waltham, MA, USA).

### 2.15 Cell proliferation assay

Proliferation was examined using CCK8 kits (MCE, NJ, USA) according to the manufacturer’s instructions. SW620 and HCT116 cells were plated in 96-well plates at a density between 500 to 1,500 cells per well. Cell proliferation was measured every 24 hours for 4 days. Absorbances were measured using a Microplate Reader (Bio-Rad, Hercules, CA, USA) at 450 nm.

### 2.16 Wound healing assay

Wound healing assays were performed using Culture-Inserts (ibidi, Martinsried, Germany), according to the manufacturer’s instructions. The cell density was adjusted to 7×105 cells/mL and 70 µl of the cell suspension was added to each Culture-Insert well. After incubating at 37°C and 5% CO2 for 24 h, culture-inserts were removed from the wells. The migration of SW620 cells was observed at 0h, 6h, and 12h, and HCT116 cells were observed at 0h, 24h, and 48h.

### 2.17 Cell invasion assays

Cell invasion assays were performed by matrigel invasion assay using transwell chambers (24-well format; BD Falcon, Franklin Lakes, NJ, USA). Matrigel (BD Biosciences, Franklin Lakes, NJ, USA) was diluted to 1 mg/ml in serum-free DMEM medium. Then add 60 µl to the upper surface of the chamber and incubate at 37 degrees for 2 hours. SW620 cells(20,000 cells/insert) were added to the chambers and incubated for 36 h, and HCT116 cells(60,000 cells/insert) were added to the chambers and incubated for 48 h. Complete medium (500 µl) was added to the lower chamber as a chemoattractant. After incubation, inserts were fixed and stained with 0.1% crystal violet solution. The number of cells was counted in 5 random microscopic fields (magnification 100 ×).

### 2.18 Statistical analysis

Statistical analysis was performed using GraphPad Prism, Version 8.0 (GraphPad, San Diego, CA, USA). All data were presented as mean ± SD. Statistical analysis was performed using one-way ANOVA followed by Dunnett’s multiple comparisons test to compare multiple groups. The asterisk symbol represents statistical difference (*P < 0.05, **P < 0.01, and ***P < 0.001. NS, not significant difference).

## 3 Results

### 3.1 Outline of the CRC spatial transcriptomics data

**Figure 1:**
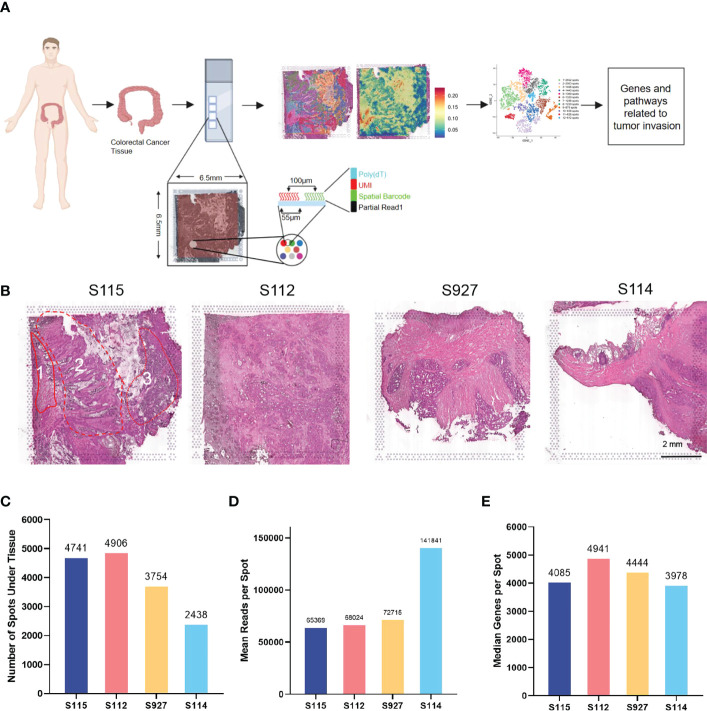
Outline of the CRC spatial transcriptomics data. **(A)** Workflow of spatial transcriptomics (ST) for CRC. CRC tissues from four patients (S115, S112, S927, S114) were used for ST by 10x Genomics Visium. **(B)** HE staining results of four samples. Sample 115 contained three parts: 1, invasive frontier; 2, cancer center area; 3, pre-cancerous area. The scar bar is 2mm. **(C)** The number of spots detected in the tissues of four samples. **(D)** The mean number of reads per spot in four samples. **(E)** The median number of genes detected in each spot.

10× Genomics spatial transcriptome for library construction has four capture regions on each slide (20). Each capture area is 6.5×6.5mm in size and contains 5000 barcode spots. The data are analyzed based on barcode information to localize every spot. In the present study, four tumor tissues containing the whole layer of the tumor tissues from the epithelial layer to the serosal layer from four CRC patients (S112, S114, S115, and S927) were analyzed (**Figure 1A**). Clinical information of the four CRC patients is shown in **Supplementary Table S1**. All the four samples had high quality RNA (**Table 1**). The H&E staining images of the four sections were shown in **Figure 1B**. The S115 section contains the pre-cancerous area, the cancer area, and the invasive frontier. The S112 contains the cancer area from the inner side to serosal layer. The remaining S927 and S114 sections mainly contain the cancer invasive frontier. The results showed that there were 4741, 4906, 3754, and 2438 spots in S115, S112, S927, and S114 sections, respectively (**Figure 1C**). The mean reactions per spot were 65369, 68024, 72715, and 141841 for each section (**Figure 1D**). The mean number of genes that were detected in each spot was respectively 4085, 4941, 4444, and 3978 (**Figure 1E**). Generally, each spot could detect 4362 genes. In total, the four samples were qualified for further analysis.

**Table 1:** The quality control of the samples.

Sample name	RIN	Concentration(μg/μl)	A260/280	A260/230	Volume (μl)	Total amount (μg)	28S/18S	Grade
S927	8.9	0.41	1.88	1.5	15	6.18	2.40	A
S115	9.3	2.76	2.03	2.11	15	41.37	3.00	A
S114	7.9	2.30	1.96	1.71	15	34.57	1.80	A
S112	7.2	4.07	1.97	1.65	15	61.11	1.70	A

### 3.2 The clustering analysis of the cancerous region among the four CRC samples

**Figure 2:**
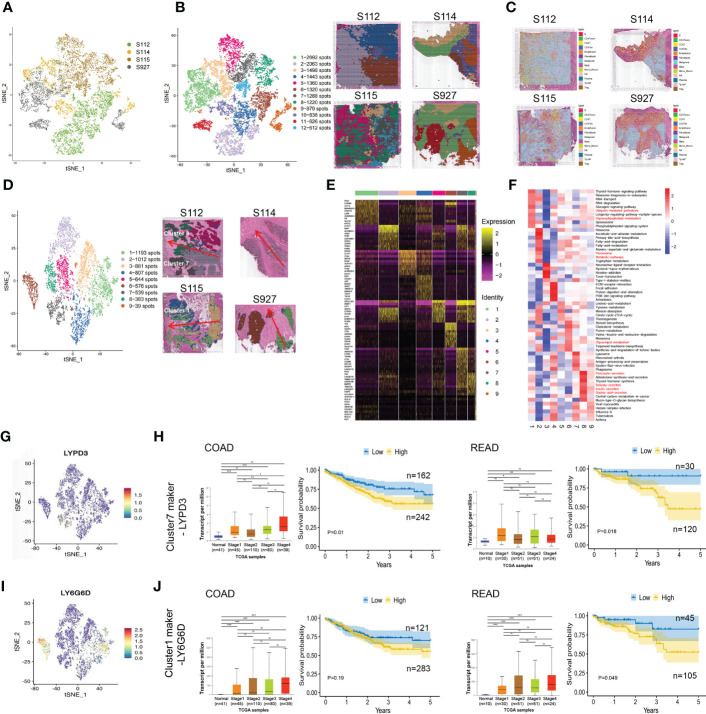
The clustering analysis of the cancerous region among the four CRC samples. **(A)** tSNE map showed the origin sites of different cell clusters from four CRC patients. **(B)** Dimensionality reduction and clustering of four samples. The left panel: tSNE map showed 12 different clusters in 4 CRC samples. The right panel: distribution of each cell cluster in tissues. **(C)** The spot map showed the composition of cell types identified in each spot on the sample slice. **(D)** Dimensionality reduction and clustering analysis of cancerous regions in the four CRC samples. Totally 9 clusters were identified. **(E)** Heatmap of the marker gene expression in the nine clusters. **(F)** Change of Signaling pathways in the nine clusters. The most obvious changed pathways along the progression were marked in red. **(G)** tSNE map showed the spatial distribution of cluster7 maker gene LYPD3 in four CRC tissues. **(H)**The relationship of LYPD3 expression level and cancer stage/progression-free survival in colon cancer (left) and rectal cancer (right) from TCGA database. **(I)** tSNE map showed the spatial distribution of cluster1 maker gene LY6G6D in four CRC tissues. **(J)** The relationship of LY6G6D expression level and cancer stage/progression-free survival in colon cancer (left) and rectal cancer (right) from TCGA database.*P < 0.05, **P < 0.01 and ***P < 0.001. NS, not significant difference.

After normalizing the data using sctransform in Seurat, PCA was used for dimensionality reduction, and t-SNE was used for clustering presentation. Since the t-SNE clustering visualization results showed a sizeable inter-sample differentiation of the clusters in the four samples, we speculated that there might be a batch effect in each sample slice (**Figure 2A**). So, we switched to the MNN algorithm to remove the batch effect and found 12 different clusters (**Figure 2B**). The cell types were then identified by Spotlight software. The blue regions were identified as cancer regions, which was generally consistent with the H&E staining observations (**Figure 2C**). But the algorithm failed to distinguish between cancer and non-cancerous regions of S112 when the cancer region was mixed with non-cancerous regions (**Figure 2C**). Furthermore, we focused on the ITH among the cancerous component outlined by the cancer pathologist. Nine different clusters were identified using the same method of dimensional clustering (**Figure 2D**). Cluster 1 was at the invasive frontiers of S115 section, while cluster 7 and 8 were at the invasive frontiers of S112 section. Subsequently, the spatially featured genes for each cluster were identified by the findmarker function (**Figure 2E**, **Table 2**).

**Table 2:** Maker gene of clusters in cancerous regions.

cluster 1	cluster 2	cluster 3	cluster 4	cluster 5	cluster 6	cluster 7	cluster 8	cluster 9
PCK1	STRIT1	PRSS2	DIO2	IGFL2−AS1	TEK	PAEP	CA4	AL591501.1
LY6G6D	BAAT	CLCA1	COL9A1	DMBT1	NEFL	ANKRD33	LINC00114	HLA−G
SYT13	AC104823.1	ITLN1	GRP	LCP1	VENTX	LYPD3	SMIM2−AS1	ECEL1
MIR4458HG	GRM1	REP15	MOXD1	ADH1C	CT83	TRPM8	LINC01186	IGFN1
CKMT1A	PPBP	REG4	WNT2	CYP4X1	ADGRG2	HTN1	BMPER	ABHD12B
LGR6	AC073365.1	ALDH1A1	TMEM119	OLFM2	LCN15	GSDMA	TM4SF20	COL28A1
AC004556.3	CPED1	AKAP3	DNM3OS	EYA1	GLRA2	EEF1A2	LINC02577	BOK−AS1
HR	CERS4	ITLN2	TCF21	BX664727.3	CDH16	NCCRP1	FABP2	ALDH1L1
SLC1A7	PIWIL1	AL365226.2	TAFA5	FFAR4	KCNT1	LINC01170	SLC16A14	MUC15
NPTX2	FIRRE	KLK12	FOXF2		GALNT14	KLK4		KLK7

By GSVA analysis, the difference between the pathway scores of each cluster and all other clusters was analyzed separately. The top 10 pathways with t-values ranked from most significant to most minor in each cluster were listed in a heatmap (**Figure 2F**). The Ubiquitin-mediated proteolysis and Glycerophospholipid metabolism were highly enriched in cluster 1, while Peroxisome, Metabolic pathways were highly enriched in cluster 7, and Glycerolipid metabolism was highly enriched in cluster 8. Finally, we analyzed the top 5 marker genes of cluster 1,7,8. One marker gene of cluster 7, LYPD3, was visualized among the clusters (**Figure 2G**, **Supplementary Figure S1A**). LYPD3 expression was increased in colon cancer but not rectal cancer tissues. But the LYPD3 expression level was associated with short progression-free survival (PFS) in both cancer types (**Figure 2H**) using the TCGA database. Similarly, the LY6G6D, marker gene of cluster 1, was up-regulated in both colon cancer and rectal cancer tissues. The LYPD3 expression level was also associated with short progression-free survival (PFS) (**Figures 2I, J**, **Supplementary Figure S1B**). Therefore, these spatially resolved transcriptomic results revealed the general ITH among cancerous regions in CRC tissues along the invasion direction.

### 3.3 The up-regulated genes in invasive clusters that were associated with prognosis of CRC patients

**Figure 3:**
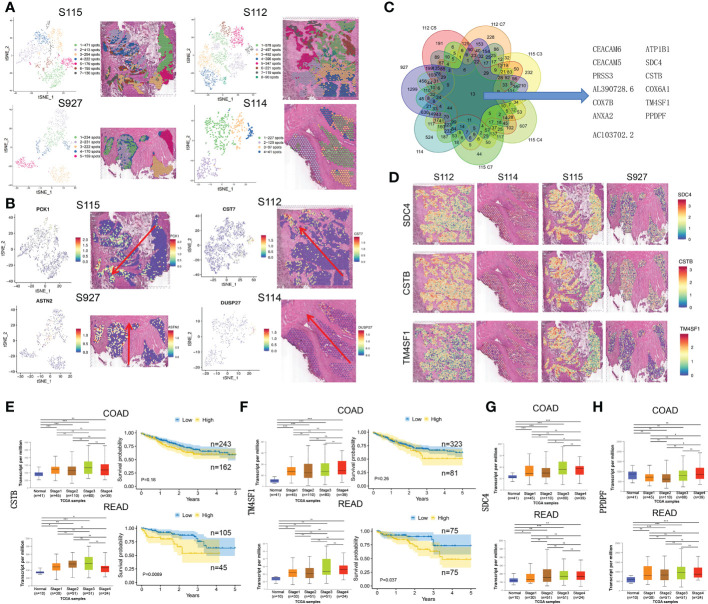
The up-regulated genes in invasive clusters that were associated with prognosis of CRC patients. **(A)** Left: four tSNE map shows the results of the dimensionality reduction and clustering analysis of the S115, S112, S114, and S927 separately. Right: the figure reflects the distribution of these clusters in the corresponding sample. **(B)** Left: the spatial distribution of the four invasive clusters**’** marker gene. Right: the visualization of expression in the corresponding samples. **(C)** Thirteen genes were screened out from the four CRC samples, which were commonly highly expressed in the invasive clusters. **(D)** Expression of SDC4 (upper panel), CSTB (middle panel), and TM4SF1 (lower panel) in four CRC samples. **(E, F)** The relationship of CSTB **(E)** and TM4SF1 **(F)** expression level and cancer stage/progression-free survival in colon cancer (up) and rectal cancer (down) from TCGA database. **(G, H)** The relationship of CSTB **(G)** and PPDPF **(H)** expression level and cancer stage in colon cancer (up) and rectal cancer (down) from TCGA database. *P < 0.05, **P < 0.01 and ***P < 0.001. NS, not significant difference.

Due to differences between the four samples, we used the same method above for dimensionality reduction and clustering of pre-annotated cancer tissue regions in the four samples separately (**Figure 3A**). At the same time, we selected the marker gene of the invasive clusters in each sample to display. PCK1, CST7, ASTN2, and DUSP27 were respectively selected as the marker gene of S115, S112, S927, and S114 and displayed in **Figure 3B**. To explore CRC invasion-associated genes, we analyzed the shared genes between the invasive clusters in each sample. These clusters include cluster 5, and 7 of S112, cluster 3, 4, and 7 of S115, and all cell clusters of S114 and S927. We found that 13 genes (CEACAM6, ATP1B1, CEACAM5, SDC4, PRSS3, CSTB, AL390728.6, COX6A1, COX7B, TM4SF1, ANXA2, PPDPF, AC103702.2) were highly expressed in all these cell clusters using Venn diagrams (**Figure 3C**). We used the Loupe Browser software to visualize the expression of these 13 genes in 4 samples (**Figure 3D**, **Supplementary Figure S2A**). The bulk expression of CSTB (**Figure 3E**) and TM4SF1 (**Figure 3F**) was associated with both advanced tumor stage and poor PFS time in both colon and rectal cancer patients. Although the expression of SDC4 and PPDPF was elevated in advanced stage tumors, their expression level was not associated with the prognosis of CRC patients (**Figures 3G, H**). Furthermore, the bulk expression of ATP1B1 was lower in advanced stage CRC tissues (**Supplementary Figure S2B**). We also found that the CRC stem cell marker EpCAM was highly expressed in the invasive frontiers (**Supplementary Figure S2C**). Taken together, we found that SDC4, CSTB, TM4SF1, ATP1B1, and PPDPF were mainly expressed in the invasive frontiers of CRC and associated with unfavorable prognosis of CRC patients.

### 3.4 Pathways and genes change with tumor progression

**Figure 4:**
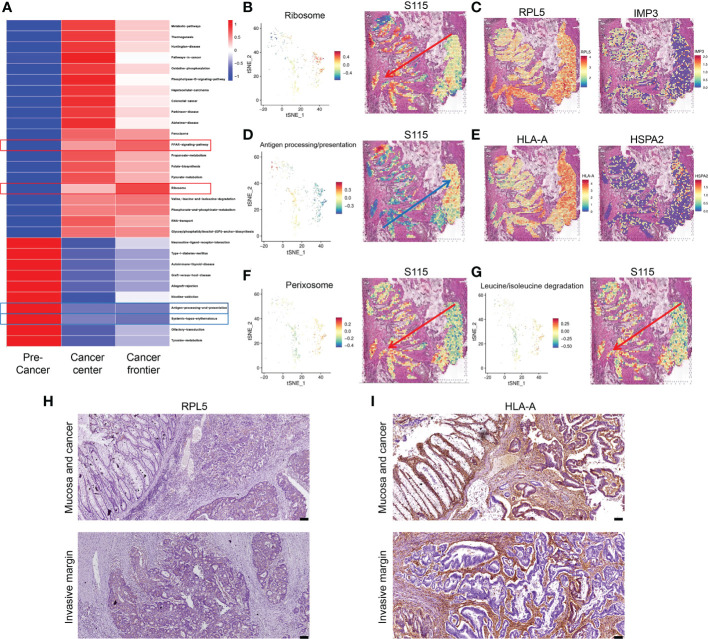
Signaling pathways and genes altered in CRC progression. **(A)** Changes in signaling pathways as CRC developed in sample S115 by GSVA analysis. **(B)** Spatial distribution and expression trend of ribosome pathway in S115. **(C)** RPL5 (left) and IMP3 (right) expression gradually increased in the direction of the red arrow. **(D)** Spatial distribution and expression trend of antigen processing and presentation pathway in S115. **(E)** HLA-A (left) and HSPA2 (right) expression gradually increased in the direction of the blue arrow. **(F, G)** Expression direction of peroxisome pathway **(F)** and leucine and isoleucine degradation pathway **(G)** in S115. **(H, I)** Immunohistochemical staining showed expression of RPL5 **(H)** and HLA-A **(I)** in Mucosa and cancer(up) and Invasive margin(down) (n=45). The scale bars on the lower right in **(H)**, **(I)** are 100 µm.

Most CRCs develop along the adenoma-precancerous lesion-cancer cascade (22). In the S115 sample, pre-cancerous lesion, cancer, and the invasive frontier can be found in the same section. We used GSVA analysis to calculate pathway activity scores between the pre-cancerous tissue, cancerous tissue, and invasive front. The results showed that some pathway scores gradually increased with the progression of CRC, such as the ribosome pathway and PPAR signaling pathway (**Figure 4A**). Conversely, the scores of some pathways gradually decreased with CRC progression, such as the antigen processing and presentation (APP) and systemic lupus erythematosus pathway (**Figure 4A**). The ribosome pathway score was shown in **Figure 4B**. It has been reported that cancer cells promote metastasis by increasing the formation of ribosomes (23). The spatial expression of RPL5 and IMP3 in ribosome pathway were gradually increased from pre-cancerous tissue to invasive frontiers (**Figure 4C**). In contrast, the APP pathway score was decreased during CRC invasion (**Figure 4D**). Tumors can escape T-cell responses by losing human leukocyte antigen (HLA) class I molecules (24). The expression of HLA-A and HSPA gradually decreased from pre-cancerous tissue to invasive frontiers (**Figure 4E**). The other pathways that were elevated along the invasion path, such as Perixosome pathway (**Figure 4F**), Leucine/isoleucine degradation (**Figure 4G**), and others (**Supplementary Figure S3**), were also visualized. We found that the expression of RPL5 was increased (**Figure 4H**), while HLA-A was downregulated (**Figure 4I**) along cancer invasion in clinical CRC samples using immunohistochemical staining. Altogether, we found that the ribosome signaling, antigen processing, and presentation pathway were gradually changed along the CRC invasion.

### 3.5 Dynamic changes of cancer progression revealed by cell trajectory and pseudo-time analysis

**Figure 5:**
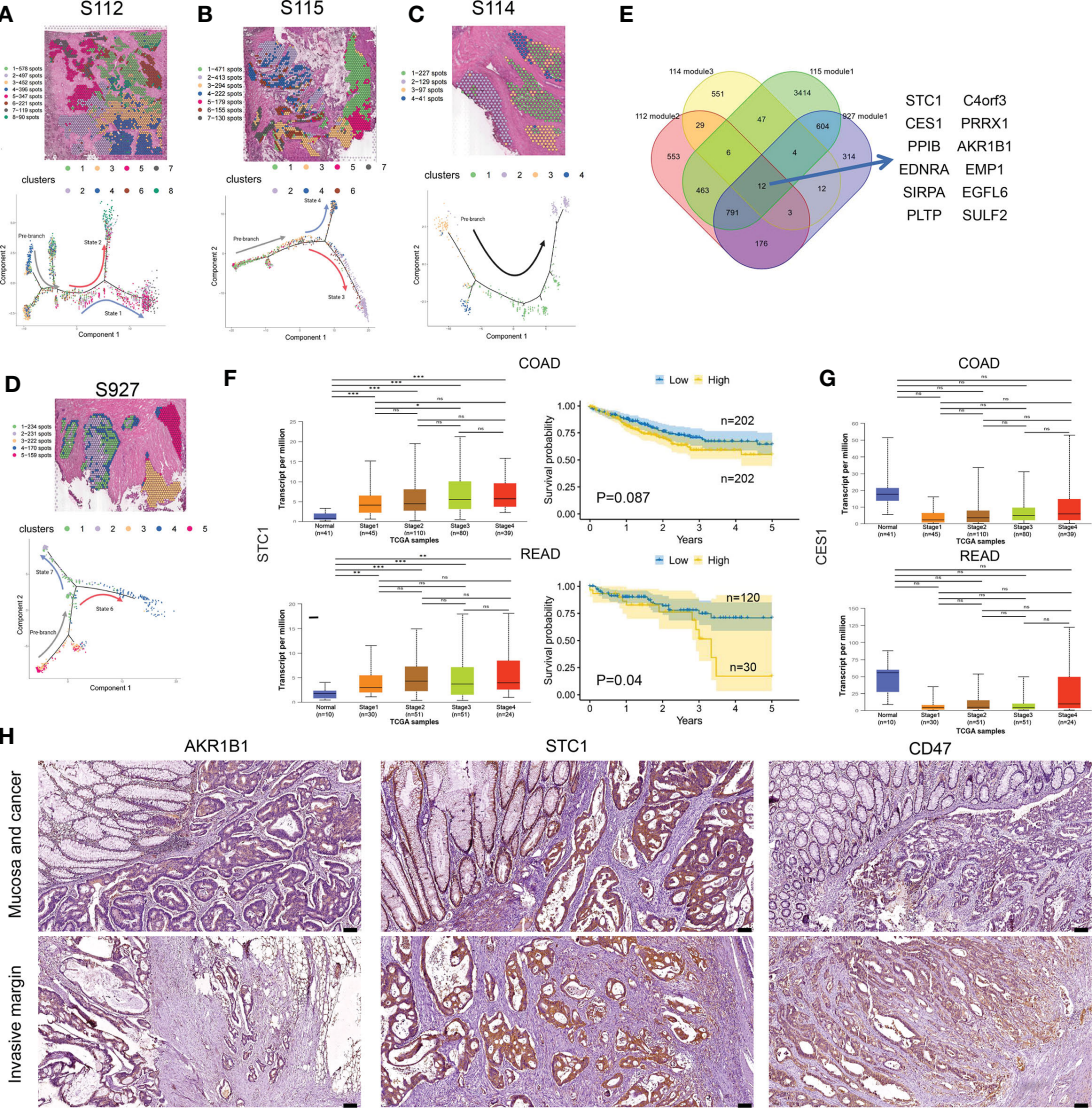
Cell differentiation trajectories in CRC obtained by pseudotime analysis. **(A–D)** tSNE map shows the results of the dimensionality reduction and clustering analysis of S112 **(A)**, S115 **(B)**, S114 **(C)**, and 927 **(D)** (up). Results of pseudotime cell trajectory in S112 **(A)**, S115 **(B)**, S114 **(C)**, and S927 **(D)** (down). **(E)** Twelve genes were screened by invasive modules. **(F)** The relationship of STC1 expression level and cancer stage/progression-free survival in colon cancer (up) and rectal cancer (down) from TCGA database. **(G)** The relationship of CES1 expression level and cancer stage in colon cancer (up) and rectal cancer (down) from TCGA database. **(H)** Immunohistochemical staining showed the expression of AKR1B1(left panel), STC1(middle panel), and CD47(right panel) in Mucosa and cancer(up) and Invasive margin(down) (n=45). The scale bars on the lower right are 100 µm. *P < 0.05, **P < 0.01 and ***P < 0.001. NS, not significant difference.

To gain further insight into the invasion of CRC, we performed pseudo-time analysis to infer differentiation trajectories during cell invasion. We independently analyzed the pre-annotated cancer tissue regions of the four samples and found different cellular differentiation trajectories in cancer tissue (**Figures 5A–D**). We obtained the dynamic change of gene expression during cell invasion. The differentially expressed genes in different modules were shown in heatmaps (**Supplementary Figures 4A–D**). The gene expression level was represented by color from blue to red, and then the genes with similar expression patterns in the developmental trajectory were clustered. We selected the modules of gradually up-regulated genes along the invasion depth in the four samples for further analysis. We performed KEGG enrichment analysis on the differentially expressed genes of the selected modules, respectively (**Supplementary Figures S4A–D**).

Then we intersected the genes of the selected modules in the four samples again and found that 12 genes existed in the intersection, including STC1, AKR1B1, SIRPA, C4orf3, EDNRA, CES1, PRRX1, EMP1, PPIB, PLTP, SULF2, and EGFL6 (**Figure 5E**). Based on the TCGA database, the bulk expression of STC1 was higher in advanced CRC tissues (**Figure 5F**). High STC1 expression was associated with poor PFS in both colon and rectal cancer patients (**Figure 5F**). The total expression of CES1 was significantly lower in advanced CRC tissues than in normal tissues, but its expression gradually increased as tumor invaded deeper (**Figure 5G**). The CD47 protein, expressed on both healthy and malignant cells, delivers a ‘don’t eat me’ signal upon binding to the SIRPA receptor on myeloid cells. Finally, we found that the expression of STC1, AKR1B1, and CD47 was increased along cancer invasion in clinical CRC samples using immunohistochemical staining (**Figure 5H**). Taken together, pseudo-time analysis confirmed the ITH in CRC tissues and meanwhile revealed novel invasion-related genes.

### 3.6 Knockdown of AKR1B1 inhibited the proliferation, migration, and invasion of CRC cells

**Figure 6:**
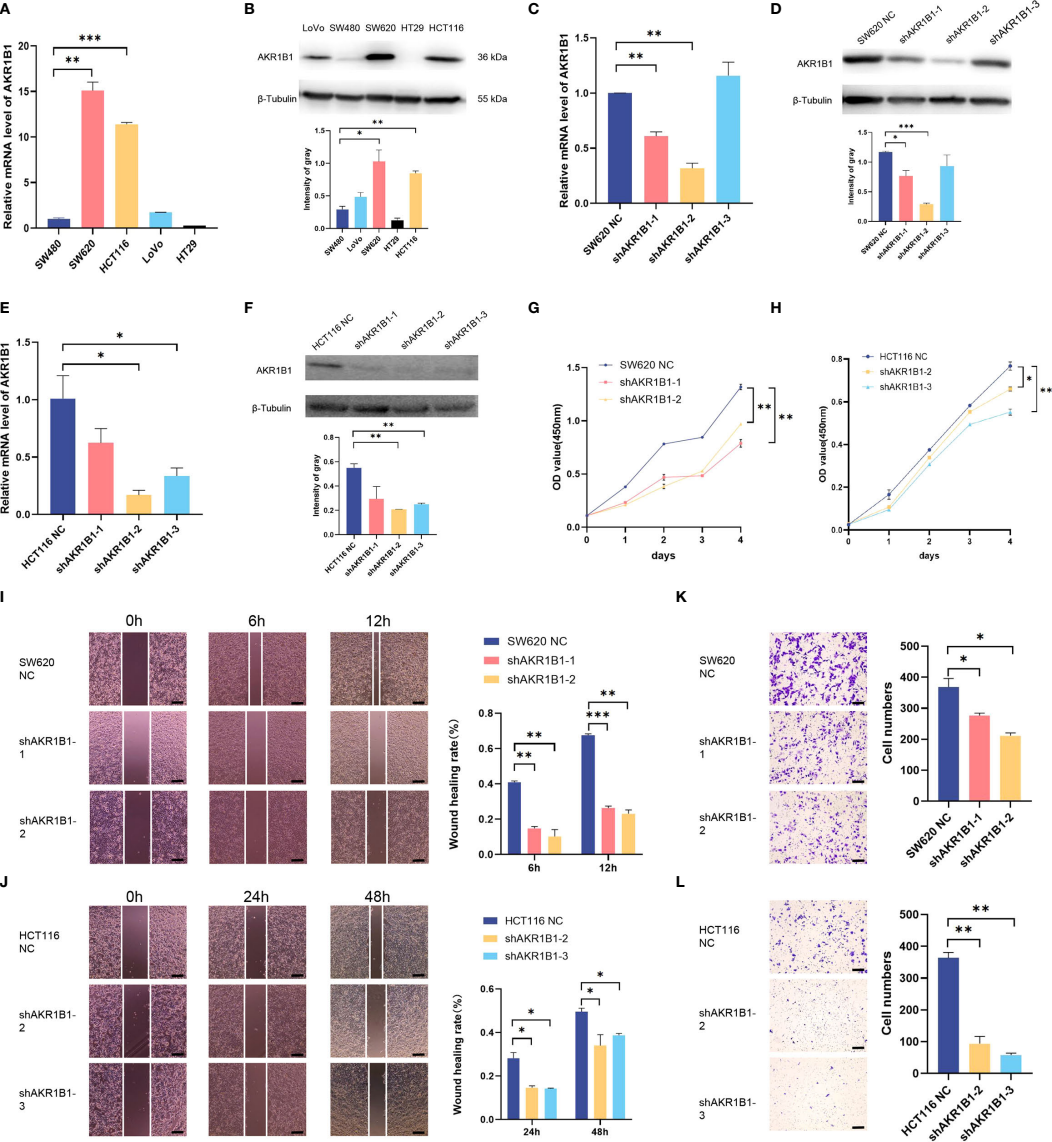
Knockdown of AKR1B1 inhibited cell proliferation, migration, and invasion. **(A, B)** The transcriptional level **(A)** and protein level **(B)** of AKR1B1 were detected in multiple colorectal cancer cell lines (SW480, SW620, HCT116, LoVo, and HT29) using RT-PCR and WB. **(C, D)** The transcriptional level **(C)** and protein level **(D)** of AKR1B1 were detected in the control and knockdown groups of SW620 using RT-PCR and WB. **(E, F)** The transcriptional level **(E)** and protein level **(F)** of AKR1B1 were detected in the control and knockdown groups of HCT116 using RT-PCR and WB. **(G, H)** Cell proliferation of SW620 **(G)** and HCT116 **(H)** in control and knockdown groups was detected using CCK8 assay. **(I, J)** Cell migration of SW620 **(I)** and HCT116 **(J)** in control and knockdown groups was detected using wound healing assay. **(K, L)** Cell invasion of SW620 **(K)** and HCT116 **(L)** in control and knockdown groups was detected using transwell assay. The scale bars on the lower right in **(I–L)** are 200 µm. *P < 0.05, **P < 0.01 and ***P < 0.001. NS, not significant difference. N=3.

Aldo-keto reductase 1 member B1 (AKR1B1), which catalyzes the reduction of prostaglandins PGH2 to PGF2α, is a major nicotinamide adenine dinucleotide phosphate reduced (NADPH)–dependent PGF synthase during arachidonic acid metabolism. AKR1B1 expression provides tumorigenic and metastatic advantage in basal-like breast cancer through activating the epithelia-mesenchymal transition and enhancing stem cell-like properties (25). Recently, AKR1B1 has been found to promote glutathione *de novo* synthesis to enhance acquired resistance to EGFR-targeted therapy in lung cancer (26). However, the role of AKR1B1 in CRC progression was poorly known. We first determined the expression level of AKR1B1 in CRC cell lines (SW480, SW620, HCT116, LoVo, and HT29) by RT-PCR and Western blot (**Figures 6A, B**). We found that AKR1B1 was highly expressed in SW620 and HCT116 cells, in which we choose to knockdown AKR1B1. The knockdown efficiency was validated by both RT-PCR and Western blot in these two cell lines (**Figures 6C–F**). We found that knockdown of AKR1B1 could remarkably suppress the proliferation of SW620 (**Figure 6G**) and HCT116 cells (**Figure 6H**). Further, the migration of SW620 and HCT116 cells using wound healing model was significantly inhibited after AKR1B1 knockdown (**Figures 6I, J**). Finally, AKR1B1 depletion also reduced the invasion of these cells (**Figures 6K, L**). In sum, AKR1B1 could promote the proliferation, migration, and invasion of CRC cells.

## 4 Discussion

This is a period of big data. We hope to provide individual treatment for the cancer patients, which relies on comprehensive knowledge of cancer biology. Recent advances in multi-omic technology provide fundamental insight into the intertumoral heterogeneity and ITH (27). We have found distinct subpopulations within the cancer cells and non-malignant cells within the cancer tissues (28). Herein, we make use of spatial transcriptomics to reveal the spatial heterogeneity along the invasion direction in CRC tissues (29). We rebuilt the gene expression status of each spatial location within the CRC tissue, established the relationship between cells, and discovered some pathways and genes related to CRC invasion.

We first picked out the cancerous regions in the four sections and found nine clusters among these regions. Due to the different pathological components in each section, we identified invasive cluster 7 and 8 in S112, and invasive cluster 1 in S115. Some of the marker genes of these clusters were associated with tumor progression, such as LYPD3 and LY6G6D. LY6G6D up-regulation was predominant in MSS CRCs characterized by an enrichment of immune suppressive regulatory T-cells and a limited repertoire of PD-1/PD-L1 immune checkpoint receptors (30, 31). Thus, LY6G6D has been used as a target for CRC therapy. On the other hand, LYPD3 protein expression was exclusively localized in primary and metastatic breast cancer tissues (32), and it was reported to maintain CRC stemness (33). We then separately analyzed the clusters in each section and found some commonly up-regulated genes in the cancer invasion margin. We found that 13 genes are highly expressed in all these cell clusters. The bulk expression of CSTB and TM4SF1 was associated with both advanced tumor stage and poor PFS time in CRC patients. TM4SF1 expression was markedly higher in CRC tissues than in non-tumor tissues and was positively correlated with poor prognosis. Downregulation of TM4SF1 inhibited the migration, invasion, and cancer stemness of CRC cells (34). CSTB belongs to the large family of cystatins and functions as an intracellular protease inhibitor. CSTB expression was inversely correlated with lung cancer stage, tumor grade, and a more unfavorable prognosis (35). We also found that the expression of SDC4 and PPDPF was elevated in advanced stage tumors, but their expression level was not associated with the prognosis of CRC patients. Our results have revealed novel genes that were closely related to CRC invasion, but their role in CRC invasion should be further elucidated, especially for CSTB, SDC4, ATP1B1, PRSS3, and PPDPF.

Most CRCs develop along the adenoma-precancerous lesion-cancer cascade (22). In the S115 sample, pre-cancerous lesion, cancer center, and invasive frontier can be found in the same section. We found that the ribosome signaling and APP pathway were gradually changed along the CRC invasion depth. It has been reported that intracellular ribosomes increased during CRC progression (23). We found that one ribosome subunit RPL5 was increased along CRC invasion. RPL5 could promote hepatocellular carcinoma cell proliferation and invasion by MDM2/P53 signaling (36). Recent studies have also revealed another ribosome component, IMP3, could promote invasion of both CRC (37) and prostate cancer cells (38). A member of APP pathway with gradually increased expression is HLA-A. It belongs to the class I molecules, which help recognition by cytotoxic T cells (39). Down-regulation of HLA-A expression correlates with a better prognosis in CRC patients (40). Immunohistochemical staining in clinical CRC tissues showed that the expression of RPL5 was increased, while HLA-A was downregulated along cancer invasion. These results implies that immune escape might facilitate CRC invasion.

Finally, pseudo-time analysis to infer differentiation trajectories during cell invasion. We found that 12 genes, including STC1, AKR1B1, SIRPA, C4orf3, EDNRA, CES1, PRRX1, EMP1, PPIB, PLTP, SULF2, and EGFL6, were gradually increased along the invasion path. Immunohistochemical staining showed that the expression of STC1, AKR1B1, and CD47 was increased along cancer invasion. AKR1B1 is a major NADPH–dependent PGF synthase to catalyze the reduction of prostaglandins PGH2 to PGF2α during arachidonic acid metabolism (25). It has been reported that AKR1B1 could provide tumorigenic and metastatic advantage in basal-like breast cancer through activating the epithelia-mesenchymal transition and enhancing stem cell-like properties (25), and targeting AKR1B1 could depress glutathione *de novo* synthesis to overcome acquired resistance to EGFR-targeted therapy in lung cancer (26). We herein reported that AKR1B1 could promote the proliferation, migration, and invasion of CRC cells, but the underlying mechanism needs further study. High STC1 expression was associated with advanced tumor stage and poor PFS in CRC patients. STC1 could promote metastasis, lipid metabolism and cisplatin chemoresistance *via* directly binding to ITGB6 in ovarian cancer (41). We also found that the total expression of CES1 gradually increased as tumor invaded deeper although its expression is higher in normal tissues. Recently, CES1 was reported to promote CRC cell survival *via* enhancing fatty acid oxidation and preventing the toxic build-up of triacylglycerols (42). Elevated CES1 expression correlated with worse outcomes in overweight patients with CRC. The other genes that were not associated with prognosis of CRC patients still have some role in cancer progression. The binding of CD47 to its receptor SIRPA delivers a ‘don’t eat me’ signal to myeloid cells (43, 44). Thus, the increased SIRPA expression could create an immunosuppressive environment in CRC margins, along with decreased HLA-A expression. EGFL6 and PPIB were reported to promote CRC invasion and drug resistance, respectively (45). Meanwhile, EDNRA, PRRX1, EMP1, AKR1B1, and SULF2 exerted an important role in cancer progression in other tumor types (25, 46–49). The role of PLTP in cancer progression was largely unknown.

According to our results, we demonstrated the spatial ITH among CRC cells, especially the genes that were up-regulated in cancer margin. Not all of these genes were associated with cancer progression based on the TCGA database. This might be caused by that the heterogeneous expression pattern of these genes. Exploring new regulators of cancer progression according to the bulk expression is not enough in the context of complex heterogeneity. The limitation of this study is that we only collected the one resected cancer tissue from four CRC patients. Furthermore, among the four samples, most of the tissues of samples S114 and S927 were on the frontier of invasion, and we could not well observe the changes in genes and pathways in the tissues of CRC patients. The high cost of spatial transcriptomic analysis and the small size of each section are the main limitations to using them in more samples. Many studies combined spatial transcriptomic with single cell transcriptomic to resolve ITH (50). However, each spot in spatial transcriptomic contains several cells, which might cause bias when combined with single cell sequencing data. One better alternative might be increasing/the revolution of spatial transcriptomic to a single cell level.

In conclusion, our spatial transcriptomic results revealed the intratumoral heterogeneity of CRC tissues and uncovered some novel genes that were associated with CRC invasion and progression.

## Data availability statement

The datasets presented in this study can be found in online repositories. The names of the repository/repositories and accession number(s) can be found in the article/**Supplementary Material**.

## Ethics statement

The study was approved by the ethical review board of the Second Affiliated Hospital of Chongqing Medical University (Chongqing China; Project identification code: 2019.133). The patients/participants provided their written informed consent to participate in this study.

## Author contributions

Conceptualization, HT-L and SY-C; Methodology, LL-P and LZhon; Resources, LZhou, SQ-L, ZJ-C, QL-W, and SY-C; Writing – Original Draft, HT-L; Writing – Review & Editing, SH and ZH-Z; Supervision, ZH-Z. All authors provided critical feedback that helped shape the research, analyses, and final manuscript. All authors have read and agreed to the published version of the manuscript.
