# Supplementary material for: Spatially resolved transcriptomics revealed local invasion-related genes in colorectal cancer
Source: Front Oncol. 2023 Feb 1;13:1089090. doi: 10.3389/fonc.2023.1089090 (PMC9928961; doi:10.3389/fonc.2023.1089090)
Supplement: Supplementary file 1 [file DataSheet_1.pdf]

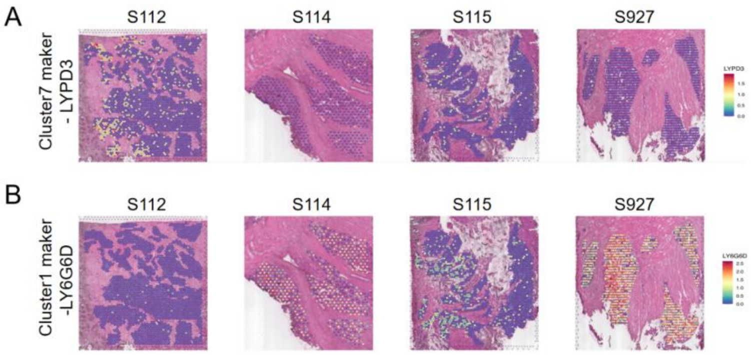

Supplementary Figure 1. Expression location Analysis of marker genes in CRC tissues. (A) Expression location of cluster7 maker gene *LYPD3* in four CRC samples. (B) Expression visualization of cluster1 maker gene *LY6G6D* in four CRC samples.

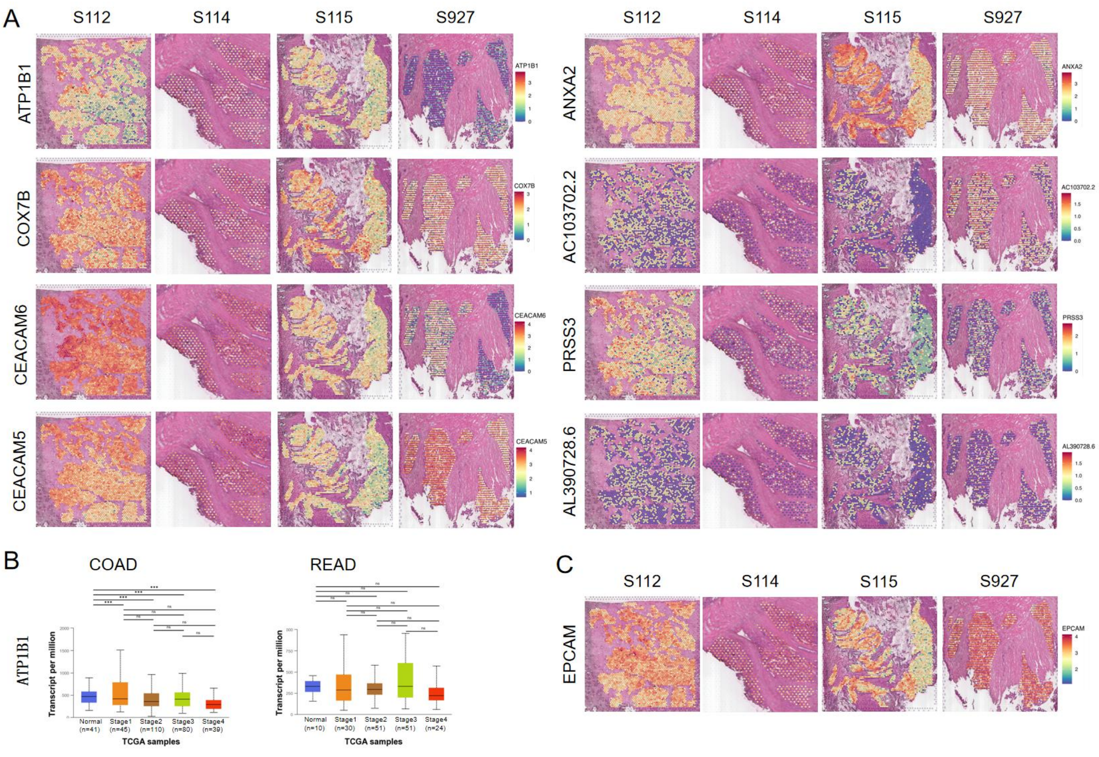

Supplementary Figure 2. Expression of invasion related genes and their relationship with prognosis. (A) Expression analysis of 8 genes (*ATP1B1, COX7B, CEACAM6, CEACAM5, ANXA2, AC103702.2, PRSS3,* and *L390728.6*) in four CRC samples. (B) The relationship of *ATP1B1* expression level and cancer stage in colon cancer (left) and rectal cancer (right) from TCGA database. (C) Expression of *EpCAM* in four samples. *P < 0.05, **P < 0.01 and ***P < 0.001. NS, not significant difference.

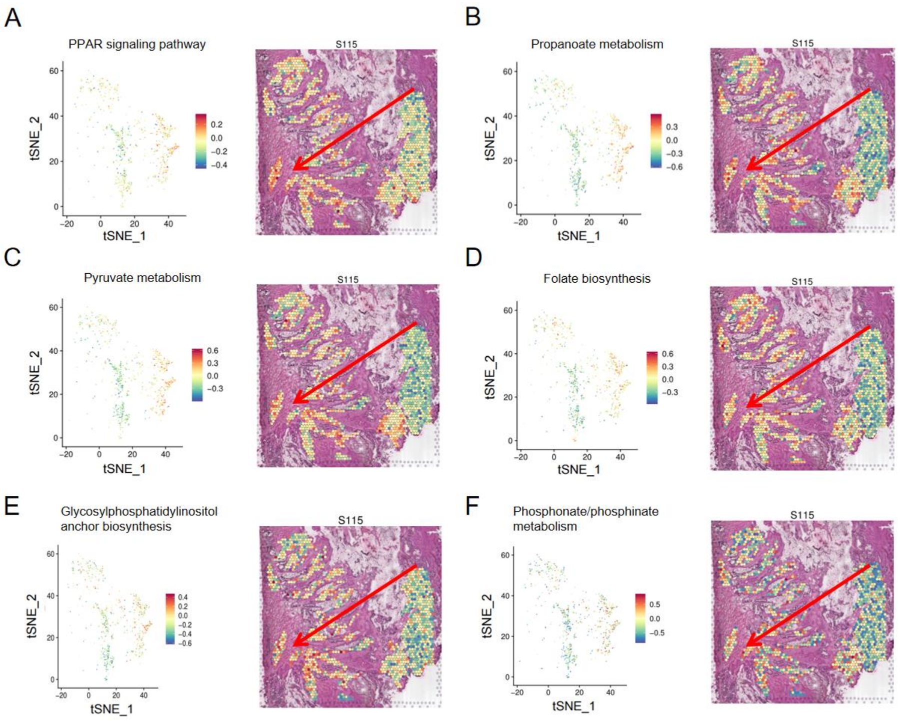

Supplementary Figure 3. Expression of signal pathways in CRC tissues. (A-F) Spatial distribution and expression trend of PPAR signaling pathway (A), propanoate metabolism pathway (B), pyruvate metabolism pathway (C), folate biosynthesis pathway (D), glycosylphosphatidylinositol anchor biosynthesis pathway (E), and phosphonate/ phosphinate metabolism pathway (F) in S115. The expression of signal pathways was enhanced as shown by the red arrow.

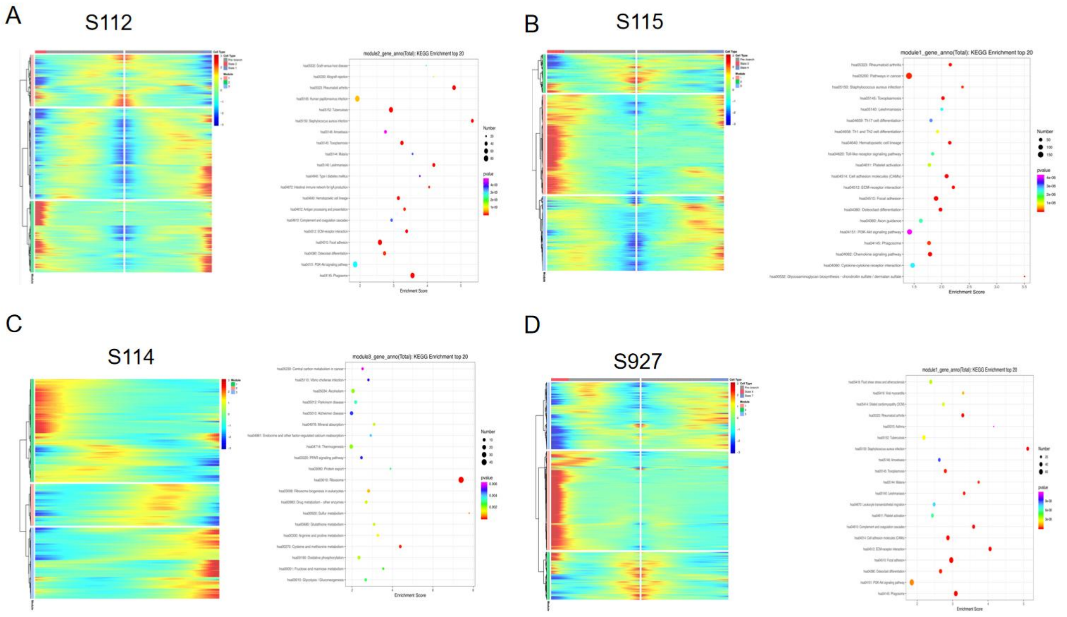

Supplementary Figure 4. Heatmap and KEGG results of invasive modules. (A-D) Heatmap of gene expression in S112 (A), S115 (B), S114 (C), and S927 (D)(left

panel). The color from blue to red indicated the gene expression from low to high. KEGG results of each selected module among which genes were up-regulated along the invasion direction in S112 (A), S115 (B), S114 (C), and S927 (D)(right panel).
